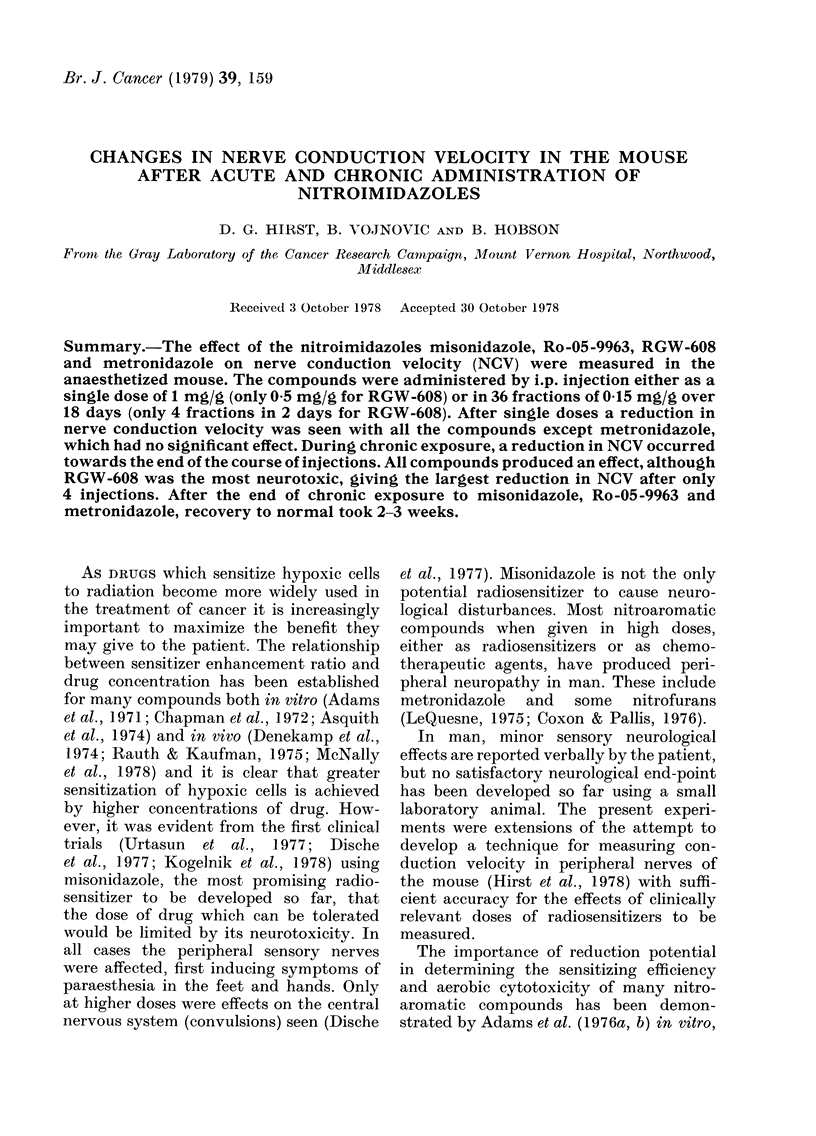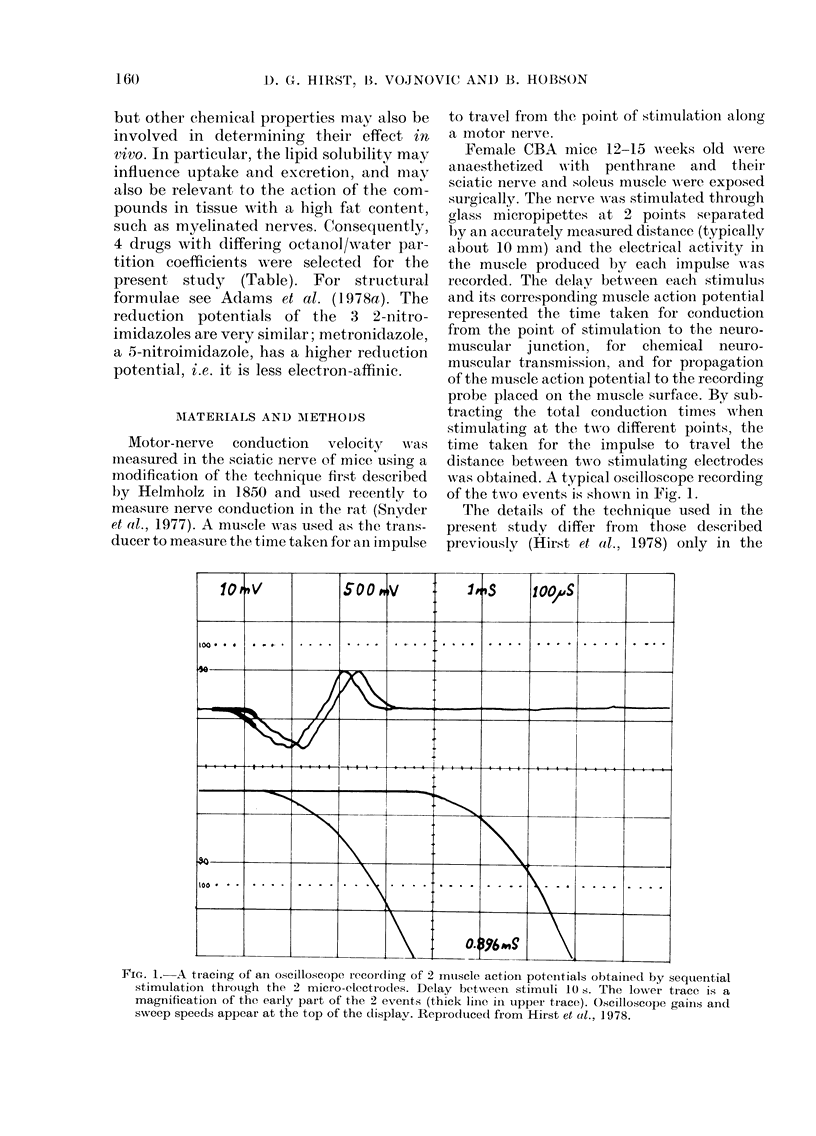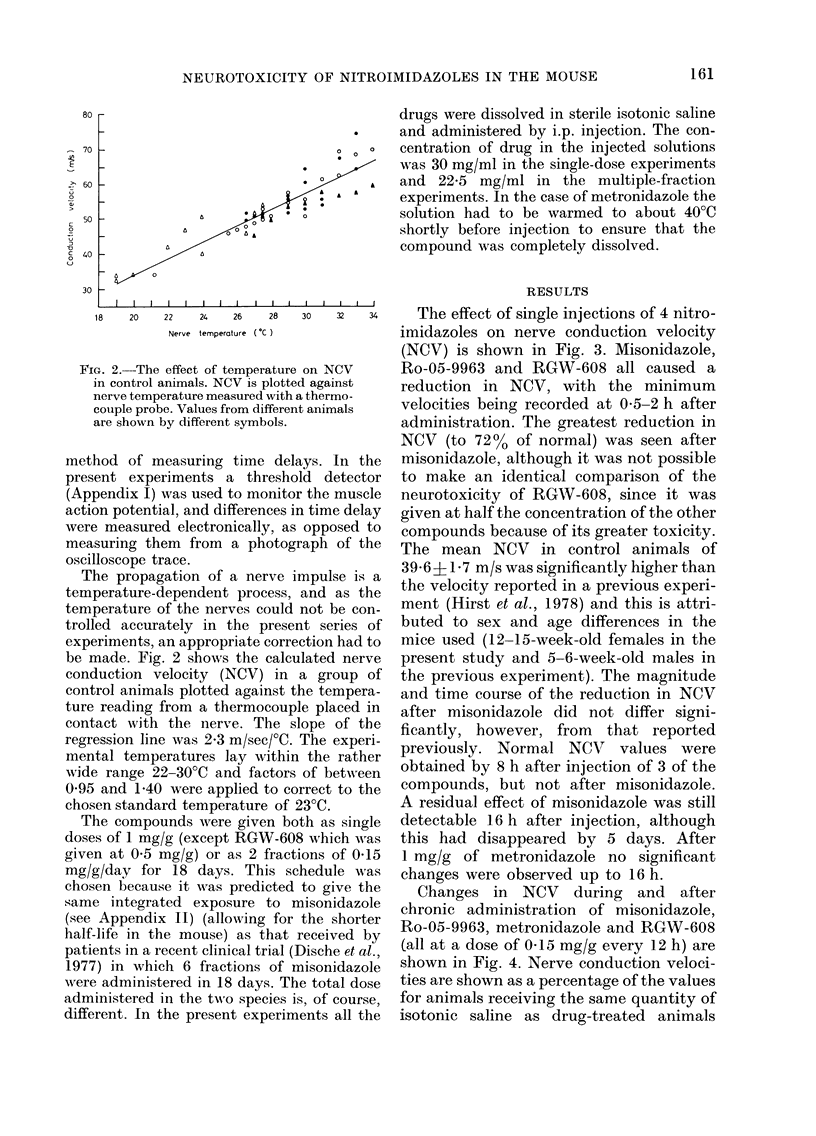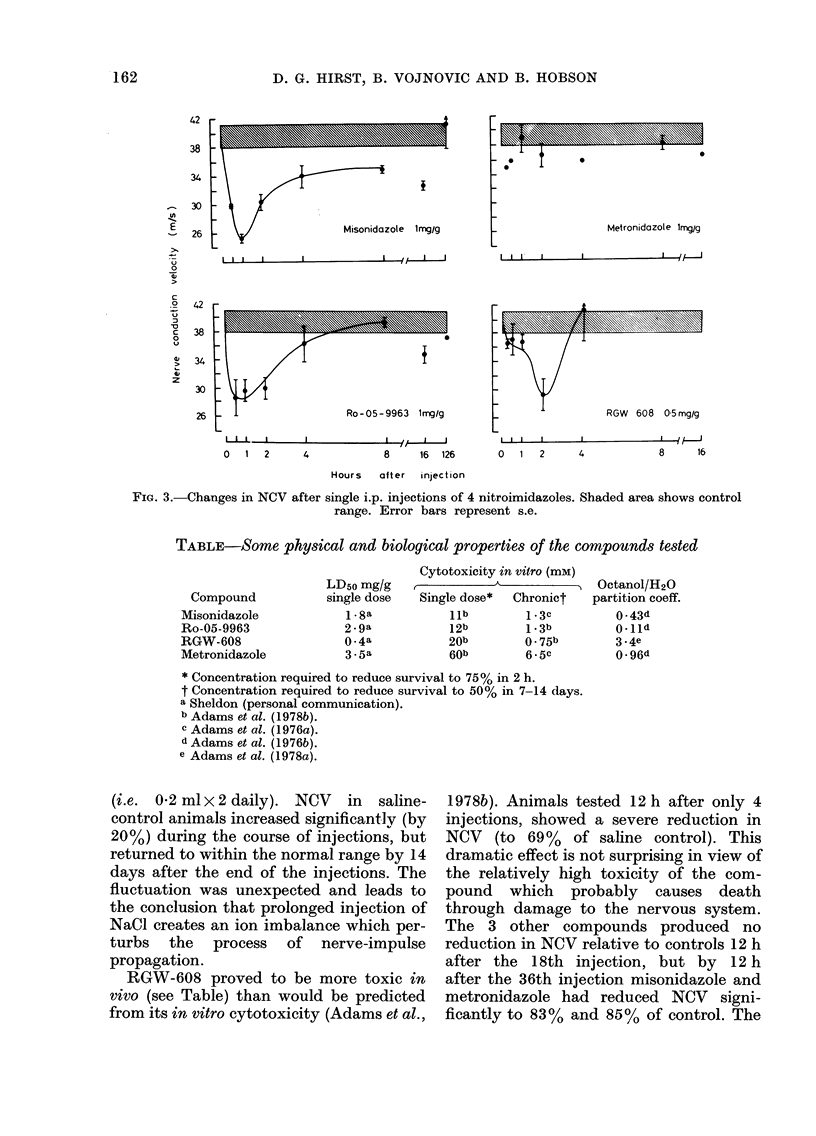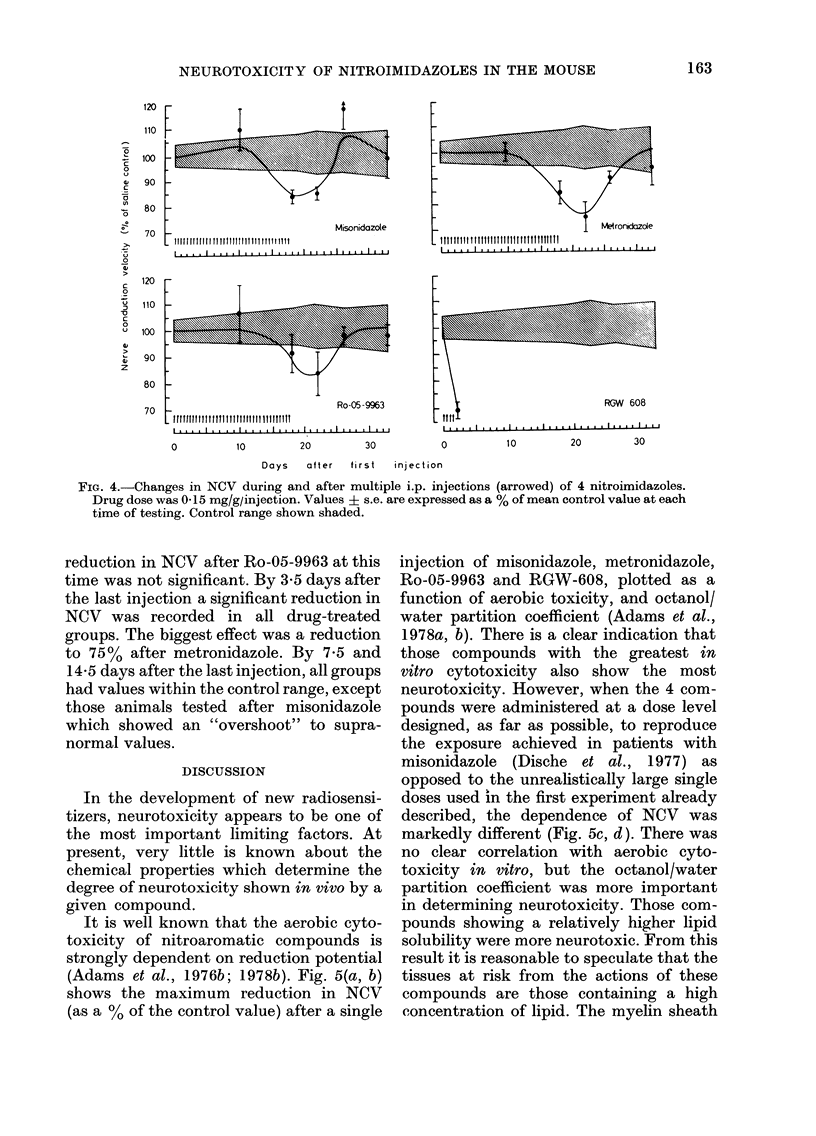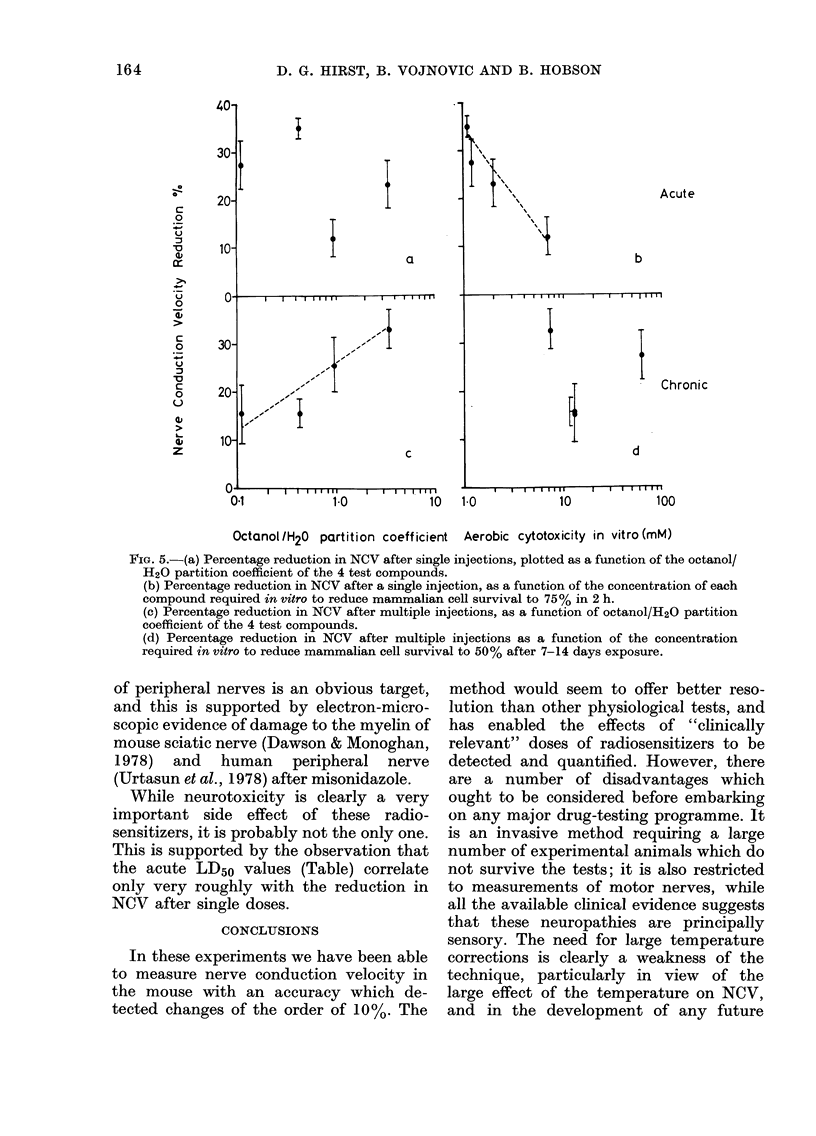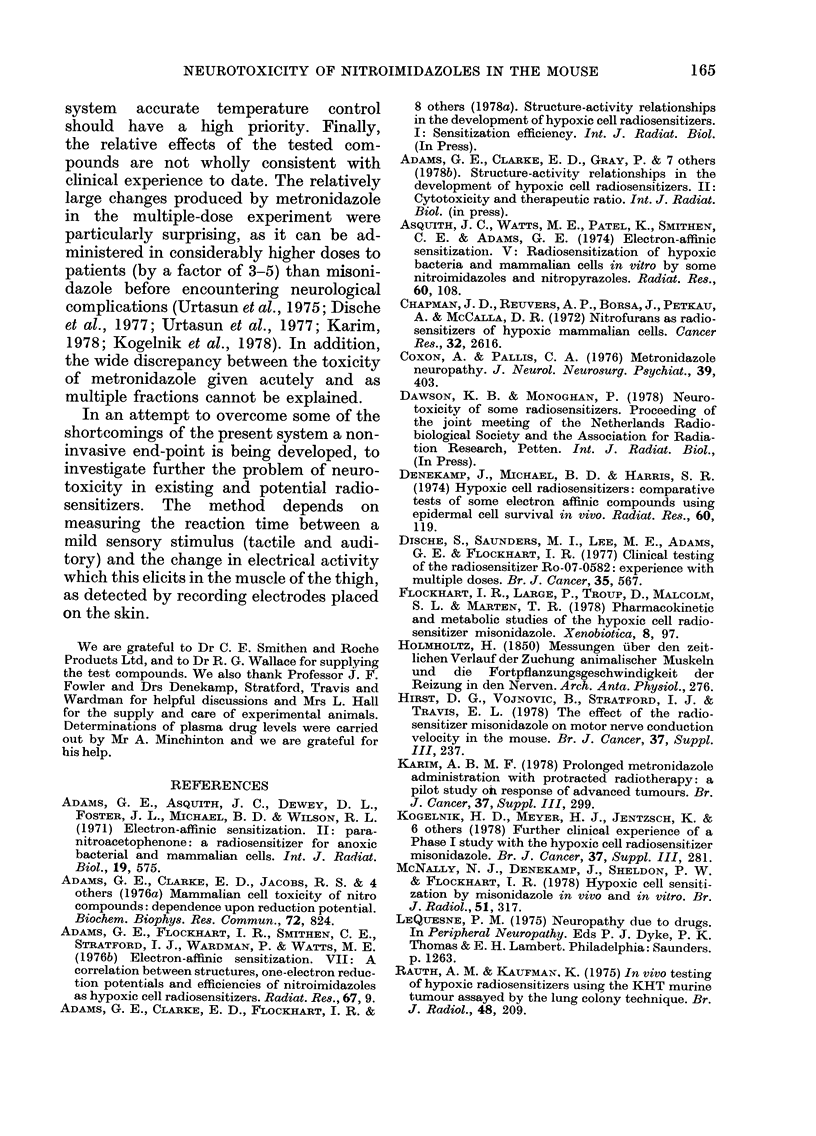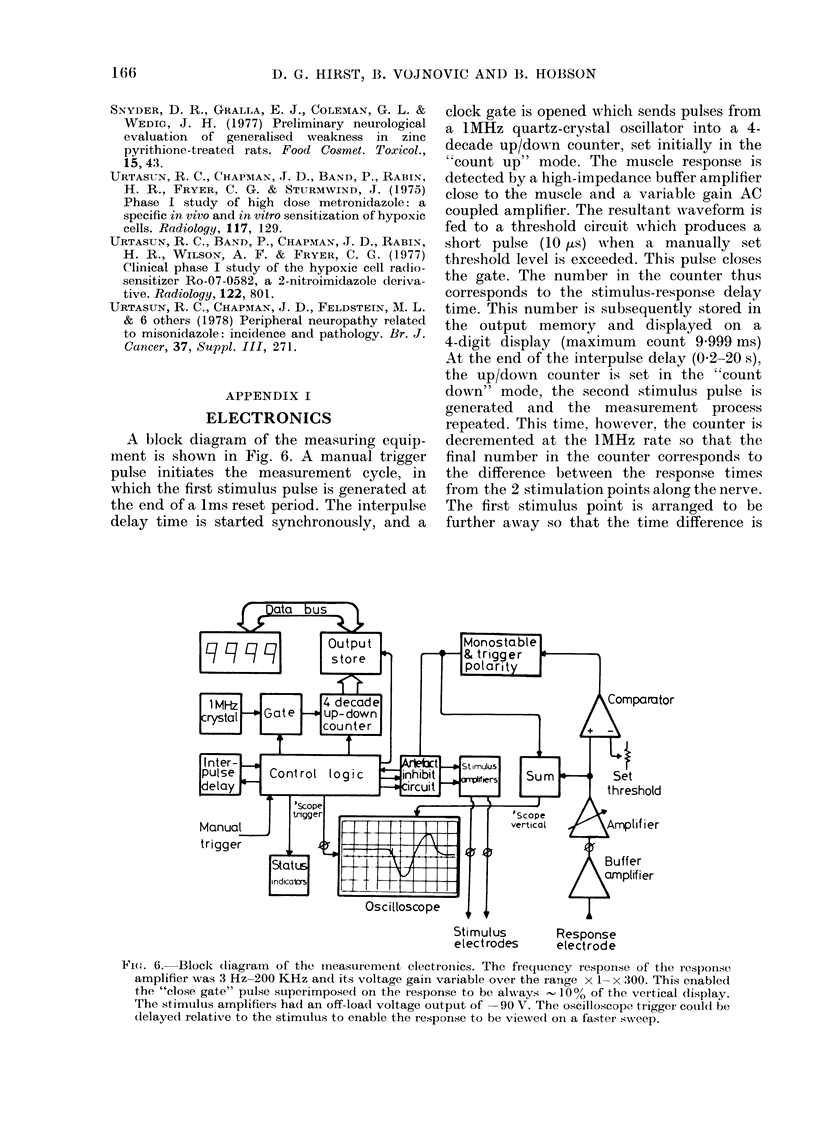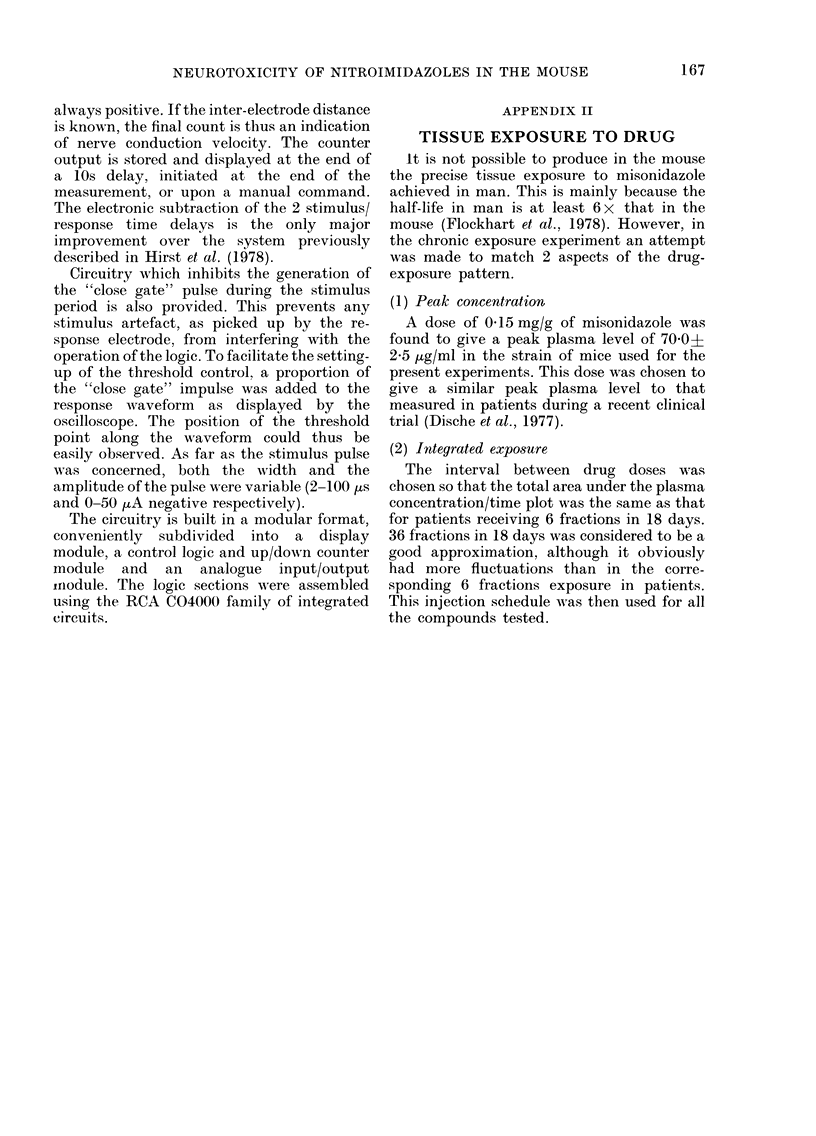# Changes in nerve conduction velocity in the mouse after acute and chronic administration of nitroimidazoles.

**DOI:** 10.1038/bjc.1979.26

**Published:** 1979-02

**Authors:** D. G. Hirst, B. Vojnovic, B. Hobson

## Abstract

The effect of the nitroimidazoles misonidazole, Ro-05-9963, RGW-608 and metronidazole on nerve conduction velocity (NCV) were measured in the anaesthetized mouse. The compounds were administered by i.p. injection either as a single dose of 1 mg/g (only 0.5 mg/g for RGW-608) or in 36 fractions of 0.15 mg/g over 18 days (only 4 fractions in 2 days for RGW-608). After single doses a reduction in nerve conduction velocity was seen with all the compounds except metronidazole, which had no significant effect. During chronic exposure, a reduction in NCV occurred towards the end of the course of injections. All compounds produced an effect, although RGW-608 was the most neurotoxic, giving the largest reduction in NCV after only 4 injections. After the end of chronic exposure to misonidazole, Ro-05-9963 and metronidazole, recovery to normal took 2-3 weeks.


*Br. J. Cancer* (1979) **39**, 159

### CHANGES IN NERVE CONDUCTION VELOCITY IN THE MOUSE AFTER ACUTE AND CHRONIC ADMINISTRATION OF NITROIMIDAZOLES

D. G. HIRST, B. VOJNOVIC AND B. HOBSON

*From the Gray Laboratory of the Cancer Research Campaign, Mount Vernon Hospital, Northwood, Middlesex*

Received 3 October 1978 Accepted 30 October 1978

**Summary.—The effect of the nitroimidazoles misonidazole, Ro-05-9963, RGW-608 and metronidazole on nerve conduction velocity (NCV) were measured in the anaesthetized mouse. The compounds were administered by i.p. injection either as a single dose of 1 mg/g (only 0·5 mg/g for RGW-608) or in 36 fractions of 0·15 mg/g over 18 days (only 4 fractions in 2 days for RGW-608). After single doses a reduction in nerve conduction velocity was seen with all the compounds except metronidazole, which had no significant effect. During chronic exposure, a reduction in NCV occurred towards the end of the course of injections. All compounds produced an effect, although RGW-608 was the most neurotoxic, giving the largest reduction in NCV after only 4 injections. After the end of chronic exposure to misonidazole, Ro-05-9963 and metronidazole, recovery to normal took 2–3 weeks.**

As DRUGS which sensitize hypoxic cells to radiation become more widely used in the treatment of cancer it is increasingly important to maximize the benefit they may give to the patient. The relationship between sensitizer enhancement ratio and drug concentration has been established for many compounds both *in vitro* (Adams *et al.*, 1971; Chapman *et al.*, 1972; Asquith *et al.*, 1974) and *in vivo* (Denekamp *et al.*, 1974; Rauth & Kaufman, 1975; McNally *et al.*, 1978) and it is clear that greater sensitization of hypoxic cells is achieved by higher concentrations of drug. However, it was evident from the first clinical trials (Urtasun *et al.*, 1977; Dische *et al.*, 1977; Kogelnik *et al.*, 1978) using misonidazole, the most promising radiosensitizer to be developed so far, that the dose of drug which can be tolerated would be limited by its neurotoxicity. In all cases the peripheral sensory nerves were affected, first inducing symptoms of paraesthesia in the feet and hands. Only at higher doses were effects on the central nervous system (convulsions) seen (Dische *et al.*, 1977). Misonidazole is not the only potential radiosensitizer to cause neurological disturbances. Most nitroaromatic compounds when given in high doses, either as radiosensitizers or as chemotherapeutic agents, have produced peripheral neuropathy in man. These include metronidazole and some nitrofurans (LeQuesne, 1975; Coxon & Pallis, 1976).

In man, minor sensory neurological effects are reported verbally by the patient, but no satisfactory neurological end-point has been developed so far using a small laboratory animal. The present experiments were extensions of the attempt to develop a technique for measuring conduction velocity in peripheral nerves of the mouse (Hirst *et al.*, 1978) with sufficient accuracy for the effects of clinically relevant doses of radiosensitizers to be measured.

The importance of reduction potential in determining the sensitizing efficiency and aerobic cytotoxicity of many nitroaromatic compounds has been demonstrated by Adams *et al.* (1976*a*, *b*) *in vitro*, but other chemical properties may also be involved in determining their effect *in vivo*. In particular, the lipid solubility may influence uptake and excretion, and may also be relevant to the action of the compounds in tissue with a high fat content, such as myelinated nerves. Consequently, 4 drugs with differing octanol/water partition coefficients were selected for the present study (Table). For structural formulae see Adams *et al.* (1978*a*). The reduction potentials of the 3 2-nitroimidazoles are very similar; metronidazole, a 5-nitroimidazole, has a higher reduction potential, *i.e.* it is less electron-affinic.

### MATERIALS AND METHODS

Motor-nerve conduction velocity was measured in the sciatic nerve of mice using a modification of the technique first described by Helmholz in 1850 and used recently to measure nerve conduction in the rat (Snyder *et al.*, 1977). A muscle was used as the transducer to measure the time taken for an impulse to travel from the point of stimulation along a motor nerve.

Female CBA mice 12–15 weeks old were anaesthetized with penthrane and their sciatic nerve and soleus muscle were exposed surgically. The nerve was stimulated through glass micropipettes at 2 points separated by an accurately measured distance (typically about 10 mm) and the electrical activity in the muscle produced by each impulse was recorded. The delay between each stimulus and its corresponding muscle action potential represented the time taken for conduction from the point of stimulation to the neuromuscular junction, for chemical neuromuscular transmission, and for propagation of the muscle action potential to the recording probe placed on the muscle surface. By subtracting the total conduction times when stimulating at the two different points, the time taken for the impulse to travel the distance between two stimulating electrodes was obtained. A typical oscilloscope recording of the two events is shown in Fig. 1.

The details of the technique used in the present study differ from those described previously (Hirst *et al.*, 1978) only in the

FIG. 1.—A tracing of an oscilloscope recording of 2 muscle action potentials obtained by sequential stimulation through the 2 micro-electrodes. Delay between stimuli 10 s. The lower trace is a magnification of the early part of the 2 events (thick line in upper trace). Oscilloscope gains and sweep speeds appear at the top of the display. Reproduced from Hirst *et al.*, 1978.

FIG. 2.—The effect of temperature on NCV in control animals. NCV is plotted against nerve temperature measured with a thermocouple probe. Values from different animals are shown by different symbols.

method of measuring time delays. In the present experiments a threshold detector (Appendix I) was used to monitor the muscle action potential, and differences in time delay were measured electronically, as opposed to measuring them from a photograph of the oscilloscope trace.

The propagation of a nerve impulse is a temperature-dependent process, and as the temperature of the nerves could not be controlled accurately in the present series of experiments, an appropriate correction had to be made. Fig. 2 shows the calculated nerve conduction velocity (NCV) in a group of control animals plotted against the temperature reading from a thermocouple placed in contact with the nerve. The slope of the regression line was 2·3 m/sec/°C. The experimental temperatures lay within the rather wide range 22–30°C and factors of between 0·95 and 1·40 were applied to correct to the chosen standard temperature of 23°C.

The compounds were given both as single doses of 1 mg/g (except RGW-608 which was given at 0·5 mg/g) or as 2 fractions of 0·15 mg/g/day for 18 days. This schedule was chosen because it was predicted to give the same integrated exposure to misonidazole (see Appendix II) (allowing for the shorter half-life in the mouse) as that received by patients in a recent clinical trial (Dische *et al.*, 1977) in which 6 fractions of misonidazole were administered in 18 days. The total dose administered in the two species is, of course, different. In the present experiments all the drugs were dissolved in sterile isotonic saline and administered by i.p. injection. The concentration of drug in the injected solutions was 30 mg/ml in the single-dose experiments and 22·5 mg/ml in the multiple-fraction experiments. In the case of metronidazole the solution had to be warmed to about 40°C shortly before injection to ensure that the compound was completely dissolved.

### RESULTS

The effect of single injections of 4 nitroimidazoles on nerve conduction velocity (NCV) is shown in Fig. 3. Misonidazole, Ro-05-9963 and RGW-608 all caused a reduction in NCV, with the minimum velocities being recorded at 0·5–2 h after administration. The greatest reduction in NCV (to 72% of normal) was seen after misonidazole, although it was not possible to make an identical comparison of the neurotoxicity of RGW-608, since it was given at half the concentration of the other compounds because of its greater toxicity. The mean NCV in control animals of 39·6 ± 1·7 m/s was significantly higher than the velocity reported in a previous experiment (Hirst *et al.*, 1978) and this is attributed to sex and age differences in the mice used (12–15-week-old females in the present study and 5–6-week-old males in the previous experiment). The magnitude and time course of the reduction in NCV after misonidazole did not differ significantly, however, from that reported previously. Normal NCV values were obtained by 8 h after injection of 3 of the compounds, but not after misonidazole. A residual effect of misonidazole was still detectable 16 h after injection, although this had disappeared by 5 days. After 1 mg/g of metronidazole no significant changes were observed up to 16 h.

Changes in NCV during and after chronic administration of misonidazole, Ro-05-9963, metronidazole and RGW-608 (all at a dose of 0·15 mg/g every 12 h) are shown in Fig. 4. Nerve conduction velocities are shown as a percentage of the values for animals receiving the same quantity of isotonic saline as drug-treated animals

FIG. 3.—Changes in NCV after single i.p. injections of 4 nitroimidazoles. Shaded area shows control range. Error bars represent s.e.

TABLE—*Some physical and biological properties of the compounds tested*

| Compound | $LD_{50}$ mg/g single dose | Cytotoxicity *in vitro* (mM) Single dose* | Chronic† | Octanol/$H_2O$ partition coeff. |
|---|---|---|---|---|
| Misonidazole | 1·8[a] | 11[b] | 1·3[c] | 0·43[d] |
| Ro-05-9963 | 2·9[a] | 12[b] | 1·3[b] | 0·11[d] |
| RGW-608 | 0·4[a] | 20[b] | 0·75[b] | 3·4[e] |
| Metronidazole | 3·5[a] | 60[b] | 6·5[c] | 0·96[d] |

* Concentration required to reduce survival to 75% in 2 h.
† Concentration required to reduce survival to 50% in 7–14 days.
[a] Sheldon (personal communication).
[b] Adams *et al.* (1978*b*).
[c] Adams *et al.* (1976*a*).
[d] Adams *et al.* (1976*b*).
[e] Adams *et al.* (1978*a*).

(*i.e.* 0·2 ml×2 daily). NCV in saline-control animals increased significantly (by 20%) during the course of injections, but returned to within the normal range by 14 days after the end of the injections. The fluctuation was unexpected and leads to the conclusion that prolonged injection of NaCl creates an ion imbalance which perturbs the process of nerve-impulse propagation.

RGW-608 proved to be more toxic *in vivo* (see Table) than would be predicted from its *in vitro* cytotoxicity (Adams *et al.*, 1978*b*). Animals tested 12 h after only 4 injections, showed a severe reduction in NCV (to 69% of saline control). This dramatic effect is not surprising in view of the relatively high toxicity of the compound which probably causes death through damage to the nervous system. The 3 other compounds produced no reduction in NCV relative to controls 12 h after the 18th injection, but by 12 h after the 36th injection misonidazole and metronidazole had reduced NCV significantly to 83% and 85% of control. The

FIG. 4.—Changes in NCV during and after multiple i.p. injections (arrowed) of 4 nitroimidazoles. Drug dose was 0·15 mg/g/injection. Values ± s.e. are expressed as a % of mean control value at each time of testing. Control range shown shaded.

reduction in NCV after Ro-05-9963 at this time was not significant. By 3·5 days after the last injection a significant reduction in NCV was recorded in all drug-treated groups. The biggest effect was a reduction to 75% after metronidazole. By 7·5 and 14·5 days after the last injection, all groups had values within the control range, except those animals tested after misonidazole which showed an "overshoot" to supra-normal values.

### DISCUSSION

In the development of new radiosensitizers, neurotoxicity appears to be one of the most important limiting factors. At present, very little is known about the chemical properties which determine the degree of neurotoxicity shown *in vivo* by a given compound.

It is well known that the aerobic cytotoxicity of nitroaromatic compounds is strongly dependent on reduction potential (Adams *et al.*, 1976*b*; 1978*b*). Fig. 5(*a*, *b*) shows the maximum reduction in NCV (as a % of the control value) after a single injection of misonidazole, metronidazole, Ro-05-9963 and RGW-608, plotted as a function of aerobic toxicity, and octanol/water partition coefficient (Adams *et al.*, 1978*a*, *b*). There is a clear indication that those compounds with the greatest *in vitro* cytotoxicity also show the most neurotoxicity. However, when the 4 compounds were administered at a dose level designed, as far as possible, to reproduce the exposure achieved in patients with misonidazole (Dische *et al.*, 1977) as opposed to the unrealistically large single doses used in the first experiment already described, the dependence of NCV was markedly different (Fig. 5*c*, *d*). There was no clear correlation with aerobic cytotoxicity *in vitro*, but the octanol/water partition coefficient was more important in determining neurotoxicity. Those compounds showing a relatively higher lipid solubility were more neurotoxic. From this result it is reasonable to speculate that the tissues at risk from the actions of these compounds are those containing a high concentration of lipid. The myelin sheath

FIG. 5.—(a) Percentage reduction in NCV after single injections, plotted as a function of the octanol/ $H_2O$ partition coefficient of the 4 test compounds.

(b) Percentage reduction in NCV after a single injection, as a function of the concentration of each compound required *in vitro* to reduce mammalian cell survival to 75% in 2 h.

(c) Percentage reduction in NCV after multiple injections, as a function of octanol/$H_2O$ partition coefficient of the 4 test compounds.

(d) Percentage reduction in NCV after multiple injections as a function of the concentration required *in vitro* to reduce mammalian cell survival to 50% after 7–14 days exposure.

of peripheral nerves is an obvious target, and this is supported by electron-microscopic evidence of damage to the myelin of mouse sciatic nerve (Dawson & Monoghan, 1978) and human peripheral nerve (Urtasun *et al.*, 1978) after misonidazole.

While neurotoxicity is clearly a very important side effect of these radiosensitizers, it is probably not the only one. This is supported by the observation that the acute $LD_{50}$ values (Table) correlate only very roughly with the reduction in NCV after single doses.

### CONCLUSIONS

In these experiments we have been able to measure nerve conduction velocity in the mouse with an accuracy which detected changes of the order of 10%. The method would seem to offer better resolution than other physiological tests, and has enabled the effects of "clinically relevant" doses of radiosensitizers to be detected and quantified. However, there are a number of disadvantages which ought to be considered before embarking on any major drug-testing programme. It is an invasive method requiring a large number of experimental animals which do not survive the tests; it is also restricted to measurements of motor nerves, while all the available clinical evidence suggests that these neuropathies are principally sensory. The need for large temperature corrections is clearly a weakness of the technique, particularly in view of the large effect of the temperature on NCV, and in the development of any future system accurate temperature control should have a high priority. Finally, the relative effects of the tested compounds are not wholly consistent with clinical experience to date. The relatively large changes produced by metronidazole in the multiple-dose experiment were particularly surprising, as it can be administered in considerably higher doses to patients (by a factor of 3–5) than misonidazole before encountering neurological complications (Urtasun *et al.*, 1975; Dische *et al.*, 1977; Urtasun *et al.*, 1977; Karim, 1978; Kogelnik *et al.*, 1978). In addition, the wide discrepancy between the toxicity of metronidazole given acutely and as multiple fractions cannot be explained.

In an attempt to overcome some of the shortcomings of the present system a non-invasive end-point is being developed, to investigate further the problem of neurotoxicity in existing and potential radiosensitizers. The method depends on measuring the reaction time between a mild sensory stimulus (tactile and auditory) and the change in electrical activity which this elicits in the muscle of the thigh, as detected by recording electrodes placed on the skin.

We are grateful to Dr C. F. Smithen and Roche Products Ltd, and to Dr R. G. Wallace for supplying the test compounds. We also thank Professor J. F. Fowler and Drs Denekamp, Stratford, Travis and Wardman for helpful discussions and Mrs L. Hall for the supply and care of experimental animals. Determinations of plasma drug levels were carried out by Mr A. Minchinton and we are grateful for his help.

### REFERENCES

ADAMS, G. E., ASQUITH, J. C., DEWEY, D. L., FOSTER, J. L., MICHAEL, B. D. & WILSON, R. L. (1971) Electron-affinic sensitization. II: para-nitroacetophenone: a radiosensitizer for anoxic bacterial and mammalian cells. *Int. J. Radiat. Biol.*, **19**, 575.

ADAMS, G. E., CLARKE, E. D., JACOBS, R. S. & 4 others (1976*a*) Mammalian cell toxicity of nitro compounds: dependence upon reduction potential. *Biochem. Biophys. Res. Commun.*, **72**, 824.

ADAMS, G. E., FLOCKHART, I. R., SMITHEN, C. E., STRATFORD, I. J., WARDMAN, P. & WATTS, M. E. (1976*b*) Electron-affinic sensitization. VII: A correlation between structures, one-electron reduction potentials and efficiencies of nitroimidazoles as hypoxic cell radiosensitizers. *Radiat. Res.*, **67**, 9.

ADAMS, G. E., CLARKE, E. D., FLOCKHART, I. R. & 8 others (1978*a*). Structure-activity relationships in the development of hypoxic cell radiosensitizers. I: Sensitization efficiency. *Int. J. Radiat. Biol.* (In Press).

ADAMS, G. E., CLARKE, E. D., GRAY, P. & 7 others (1978*b*). Structure-activity relationships in the development of hypoxic cell radiosensitizers. II: Cytotoxicity and therapeutic ratio. *Int. J. Radiat. Biol.* (in press).

ASQUITH, J. C., WATTS, M. E., PATEL, K., SMITHEN, C. E. & ADAMS, G. E. (1974) Electron-affinic sensitization. V: Radiosensitization of hypoxic bacteria and mammalian cells *in vitro* by some nitroimidazoles and nitropyrazoles. *Radiat. Res.*, **60**, 108.

CHAPMAN, J. D., REUVERS, A. P., BORSA, J., PETKAU, A. & MCCALLA, D. R. (1972) Nitrofurans as radiosensitizers of hypoxic mammalian cells. *Cancer Res.*, **32**, 2616.

COXON, A. & PALLIS, C. A. (1976) Metronidazole neuropathy. *J. Neurol. Neurosurg. Psychiat.*, **39**, 403.

DAWSON, K. B. & MONOGHAN, P. (1978) Neurotoxicity of some radiosensitizers. Proceeding of the joint meeting of the Netherlands Radiobiological Society and the Association for Radiation Research, Petten. *Int. J. Radiat. Biol.*, (In Press).

DENEKAMP, J., MICHAEL, B. D. & HARRIS, S. R. (1974) Hypoxic cell radiosensitizers: comparative tests of some electron affinic compounds using epidermal cell survival *in vivo*. *Radiat. Res.*, **60**, 119.

DISCHE, S., SAUNDERS, M. I., LEE, M. E., ADAMS, G. E. & FLOCKHART, I. R. (1977) Clinical testing of the radiosensitizer Ro-07-0582: experience with multiple doses. *Br. J. Cancer*, **35**, 567.

FLOCKHART, I. R., LARGE, P., TROUP, D., MALCOLM, S. L. & MARTEN, T. R. (1978) Pharmacokinetic and metabolic studies of the hypoxic cell radiosensitizer misonidazole. *Xenobiotica*, **8**, 97.

HOLMHOLTZ, H. (1850) Messungen über den zeitlichen Verlauf der Zuchung animalischer Muskeln und die Fortpflanzungsgeschwindigkeit der Reizung in den Nerven. *Arch. Anta. Physiol.*, 276.

HIRST, D. G., VOJNOVIC, B., STRATFORD, I. J. & TRAVIS, E. L. (1978) The effect of the radiosensitizer misonidazole on motor nerve conduction velocity in the mouse. *Br. J. Cancer*, **37**, *Suppl. III*, 237.

KARIM, A. B. M. F. (1978) Prolonged metronidazole administration with protracted radiotherapy: a pilot study on response of advanced tumours. *Br. J. Cancer*, **37**, *Suppl. III*, 299.

KOGELNIK, H. D., MEYER, H. J., JENTZSCH, K. & 6 others (1978) Further clinical experience of a Phase I study with the hypoxic cell radiosensitizer misonidazole. *Br. J. Cancer*, **37**, *Suppl. III*, 281.

MCNALLY, N. J., DENEKAMP, J., SHELDON, P. W. & FLOCKHART, I. R. (1978) Hypoxic cell sensitization by misonidazole *in vivo* and *in vitro*. *Br. J. Radiol.*, **51**, 317.

LEQUESNE, P. M. (1975) Neuropathy due to drugs. In *Peripheral Neuropathy*. Eds P. J. Dyke, P. K. Thomas & E. H. Lambert. Philadelphia: Saunders. p. 1263.

RAUTH, A. M. & KAUFMAN, K. (1975) *In vivo* testing of hypoxic radiosensitizers using the KHT murine tumour assayed by the lung colony technique. *Br. J. Radiol.*, **48**, 209.

SNYDER, D. R., GRALLA, E. J., COLEMAN, G. L. & WEDIG, J. H. (1977) Preliminary neurological evaluation of generalised weakness in zinc pyrithione-treated rats. *Food Cosmet. Toxicol.*, **15**, 43.
URTASUN, R. C., CHAPMAN, J. D., BAND, P., RABIN, H. R., FRYER, C. G. & STURMWIND, J. (1975) Phase I study of high dose metronidazole: a specific *in vivo* and *in vitro* sensitization of hypoxic cells. *Radiology*, **117**, 129.
URTASUN, R. C., BAND, P., CHAPMAN, J. D., RABIN, H. R., WILSON, A. F. & FRYER, C. G. (1977) Clinical phase I study of the hypoxic cell radiosensitizer Ro-07-0582, a 2-nitroimidazole derivative. *Radiology*, **122**, 801.
URTASUN, R. C., CHAPMAN, J. D., FELDSTEIN, M. L. & 6 others (1978) Peripheral neuropathy related to misonidazole: incidence and pathology. *Br. J. Cancer*, **37**, *Suppl. III*, 271.

APPENDIX I

### ELECTRONICS

A block diagram of the measuring equipment is shown in Fig. 6. A manual trigger pulse initiates the measurement cycle, in which the first stimulus pulse is generated at the end of a 1ms reset period. The interpulse delay time is started synchronously, and a clock gate is opened which sends pulses from a 1MHz quartz-crystal oscillator into a 4-decade up/down counter, set initially in the "count up" mode. The muscle response is detected by a high-impedance buffer amplifier close to the muscle and a variable gain AC coupled amplifier. The resultant waveform is fed to a threshold circuit which produces a short pulse (10 $\mu$s) when a manually set threshold level is exceeded. This pulse closes the gate. The number in the counter thus corresponds to the stimulus-response delay time. This number is subsequently stored in the output memory and displayed on a 4-digit display (maximum count 9·999 ms) At the end of the interpulse delay (0·2–20 s), the up/down counter is set in the "count down" mode, the second stimulus pulse is generated and the measurement process repeated. This time, however, the counter is decremented at the 1MHz rate so that the final number in the counter corresponds to the difference between the response times from the 2 stimulation points along the nerve. The first stimulus point is arranged to be further away so that the time difference is

FIG. 6.—Block diagram of the measurement electronics. The frequency response of the response amplifier was 3 Hz–200 KHz and its voltage gain variable over the range $\times 1-\times 300$. This enabled the "close gate" pulse superimposed on the response to be always $\sim 10\%$ of the vertical display. The stimulus amplifiers had an off-load voltage output of $-90$ V. The oscilloscope trigger could be delayed relative to the stimulus to enable the response to be viewed on a faster sweep.

always positive. If the inter-electrode distance is known, the final count is thus an indication of nerve conduction velocity. The counter output is stored and displayed at the end of a 10s delay, initiated at the end of the measurement, or upon a manual command. The electronic subtraction of the 2 stimulus/response time delays is the only major improvement over the system previously described in Hirst *et al.* (1978).

Circuitry which inhibits the generation of the "close gate" pulse during the stimulus period is also provided. This prevents any stimulus artefact, as picked up by the response electrode, from interfering with the operation of the logic. To facilitate the setting-up of the threshold control, a proportion of the "close gate" impulse was added to the response waveform as displayed by the oscilloscope. The position of the threshold point along the waveform could thus be easily observed. As far as the stimulus pulse was concerned, both the width and the amplitude of the pulse were variable (2–100 $\mu$s and 0–50 $\mu$A negative respectively).

The circuitry is built in a modular format, conveniently subdivided into a display module, a control logic and up/down counter module and an analogue input/output module. The logic sections were assembled using the RCA CO4000 family of integrated circuits.

APPENDIX II

### TISSUE EXPOSURE TO DRUG

It is not possible to produce in the mouse the precise tissue exposure to misonidazole achieved in man. This is mainly because the half-life in man is at least 6$\times$ that in the mouse (Flockhart *et al.*, 1978). However, in the chronic exposure experiment an attempt was made to match 2 aspects of the drug-exposure pattern.

(1) *Peak concentration*

A dose of 0·15 mg/g of misonidazole was found to give a peak plasma level of 70·0$\pm$2·5 $\mu$g/ml in the strain of mice used for the present experiments. This dose was chosen to give a similar peak plasma level to that measured in patients during a recent clinical trial (Dische *et al.*, 1977).

(2) *Integrated exposure*

The interval between drug doses was chosen so that the total area under the plasma concentration/time plot was the same as that for patients receiving 6 fractions in 18 days. 36 fractions in 18 days was considered to be a good approximation, although it obviously had more fluctuations than in the corresponding 6 fractions exposure in patients. This injection schedule was then used for all the compounds tested.